# Molecular Phylogeography and Ecological Niche Modeling of *Sibbaldia procumbens* s.l. (Rosaceae)

**DOI:** 10.3389/fgene.2019.00201

**Published:** 2019-03-13

**Authors:** Hua-Jie Zhang, Tao Feng, Jacob B. Landis, Tao Deng, Xu Zhang, Ai-Ping Meng, Hang Sun, Heng-Chang Wang, Yan-Xia Sun

**Affiliations:** ^1^CAS Key Laboratory of Plant Germplasm Enhancement and Specialty Agriculture, Wuhan Botanical Garden, Chinese Academy of Sciences, Wuhan, China; ^2^University of Chinese Academy of Sciences, Chinese Academy of Sciences, Beijing, China; ^3^Department of Botany and Plant Sciences, University of California, Riverside, Riverside, CA, United States; ^4^Key Laboratory for Plant Diversity and Biogeography of East Asia, Kunming Institute of Botany, Chinese Academy of Sciences, Kunming, China

**Keywords:** *Sibbaldia procumbens* s.l., chloroplast regions, phylogeography, ecological niche modeling, Qinghai-Tibet Plateau

## Abstract

The phylogeographical analysis and ecological niche modeling (ENM) of the widely distributed Northern Hemisphere *Sibbaldia procumbens* s.l. can help evaluate how tectonic motion and climate change helped shape the current distribution patterns of this species. Three chloroplast regions (the *atpI-atpH* and *trnL-trnF* intergenic spacers and the *trnL* intron) were obtained from 332 (156 from present study and 176 from the previous study) individuals of *S. procumbens* s.l. An unrooted haplotype network was constructed using the software NETWORK, while BEAST was used to estimate the divergence times among haplotypes. ENM was performed by MAXENT to explore the historical dynamic distribution of *S. procumbens* s.l. The haplotype distribution demonstrates significant phylogeographical structure (*N*_ST_ > *G*_ST_; *P* < 0.01). The best partitioning of genetic diversity by SAMOVA produced three groups, while the time to the most recent common ancestor of all haplotypes was estimated to originate during the Miocene, with most of the haplotype diversity having occurred during the Quaternary. The MAXENT analysis showed *S. procumbens* s.l. had a wider distribution range during the last glacial maximum and a narrower distribution range during the last interglacial, with predictions into the future showing the distribution range of *S. procumbens* s.l. shrinking.

## Introduction

Intercontinental disjunct distributions of flowering plants are believed to result from the opening and closing of land bridges (Beringia and the North Atlantic) between Eurasia and North America, with both vicariance and long-distance dispersal proposed to play important roles in shaping intercontinental disjunctions (Boufford and Spongberg, 1983; Wen and Shi, 1999; Manos and Donoghue, 2001; Ivanov et al., 2002; Riggins and Seigler, 2012). During the period of about 15–5.5 Ma, due to persistent cooling and aridification, the Beringia was hypothesized to act as the land connection between North America and Eurasia allowing floristic exchange (Riggins and Seigler, 2012; Krasnov et al., 2015). The Bering Strait disappeared during the late Miocene, and reemerged multiple times during Pleistocene glacial periods when sea levels dropped (Marincovich and Gladenkov, 1999; Gladenkov et al., 2002). In comparison, the North Atlantic land bridge is thought to have been the connection between eastern North America and northwestern Europe across Greenland during the late Eocene and Plio-Pleistocene (Qian and Ricklefs, 2000; Ian Milne, 2006).

Large-scale shifts of distribution range are believed to have taken place in response to the development of Quaternary continental ice-sheets, especially the cycles of glacial and interglacial during the Pleistocene (Ujiié and Ujiié, 1999; Zhang et al., 2007; Yang et al., 2008). Many mountain ranges have been strongly glaciated during the Quaternary ice age, the ice-free mountain top and the edge of ice sheet offering the only refugia for alpine plants (Briner et al., 2003; Provan and Bennett, 2008). The refugia were characterized with special, buffering environments, in which species can persist one or more glacial–interglacial cycles (Rowe et al., 2004; Provan and Bennett, 2008). Re-colonization in post-glacial regions may have occurred either through long-distance dispersal from unglaciated areas or from local refugia. Both ways have played important roles in shaping the distribution patterns of species. If dispersal was the predominant mechanism of re-colonization, the species would tolerate a wide range of environmental conditions (Martin et al., 2009). When species survived in local refugia, a certain relict component of this species should exist, mostly endemic and likely with a narrower tolerance range (Martin et al., 2009). High mountain ranges were especially significant for the long-term survival of plants because their height offered sufficient scope for altitudinal shifts when climate changed (Meng et al., 2007; Fu et al., 2016).

The Qinghai-Tibet Plateau (QTP) is the highest and largest plateau in the world, which is the result of collisions between the India and Europe–Asia continents (Dupont-Nivet et al., 2010; Herzschuh et al., 2010). The QTP and adjacent regions harbor hotspots of plant diversity (Myers et al., 2000; Zheng et al., 2002). Abundant precipitation and mountains in this area provide favorable conditions for the formation and differentiation of species (Meng et al., 2007; Ye et al., 2012; Favre et al., 2015). Many plants with intercontinental disjunct distributions have been suggested to originate in the QTP and adjacent regions [e.g., the genus *Kelloggia* (Nie et al., 2005), *Pistacia* (Xie et al., 2014), *Rhodiola* (Zhang et al., 2014), *Achlys japonica*–*Achlys triphylla* and *Epimedium–Vancouveria* of Berberidaceae (Nie et al., 2005; Wang et al., 2007; Zhang et al., 2007), and sect. *Quinquefoliae* (Hao et al., 2015)].

The genus *Sibbaldia* (Rosaceae) is widely distributed in Eurasia and North America, and the circumscription of this genus has long been controversial (Muravjova, 1936; Dixit and Panigrahi, 1981; Rajput et al., 1997; Eriksson et al., 2015; Feng et al., 2017). Initially, more than 40 species were included in the genus, but only 10–15 species were kept according to revisions based on morphological characters (Dixit and Panigrahi, 1981; Rajput et al., 1997; Li et al., 2003). More recently, molecular analysis revealed *Sibbaldia* in the traditional sense is polyphyletic, with the following circumscriptions occurring: *S. adpressa* and *S. sericea* were classified in *Sibbaldianthe* (Rosaceae); *S. perpusilloides* was included in *Chamaecallis* (Rosaceae); *S. micropetala* and *S. phanerophlebia* were included in *Argentina* (Rosaceae). *S. tenuis, S. pentaphylla, S. tetrandra, S. purpurea, S. omeiensis*, and *S. sikkimensis* were transferred to *Potentilla* (Rosaceae) (Eriksson et al., 2015; Feng et al., 2017). Finally, *Sibbaldia* sensu stricto is a small group centered on *Sibbaldia procumbens* s.l. including *Sibbaldia cuneata, Sibbaldia parviflora*, and *Sibbaldia semiglabra* (Rajput et al., 1997; Eriksson et al., 2015; Feng et al., 2017), which presents the intercontinental disjunct distribution between North America and Eurasia.

In the present study, we newly sequenced three chloroplast non-coding regions (*atpI*-*atpH* spacer, *trnL* intron, and *trnL*-*trnF* spacer) from 156 *S. procumbens* s.l. individuals primarily in Eastern Asia, in combination with sequences from populations in North America and Europe (Allen et al., 2015), with the goal (1) to examine the genetic structure of *S. procumbens* s.l. in the Northern Hemisphere and (2) to investigate the possible historical events that helped shape the current geographical and genetic distribution patterns of *S. procumbens* s.l.

## Materials and Methods

### Population Sampling, DNA Isolation, Amplification, and Sequencing

A total of 156 individuals representing 29 populations of *S. procumbens* s.l. were newly collected from China, Armenia, Austria, Russia, and Japan (Table 1), which covers nearly the entire geographical range of *S. procumbens* s.l. in Eurasia. Fresh leaves were collected from each individual and dried with silica gel. Total genomic DNA was extracted using the Plant Genomic DNA extraction kit (TIANGEN, Beijing, China).

**Table 1:** Genebank information of sequences used in the current study.

Species	Voucher	Haplotype ID	Genebank ID (*atpI*-*atpH*/*trnL* + *trnL*-*trnF*)
*Sibbaldianthe adpressa*	KL Marr 203973		KP965832/KP965857
*Potentilla purpurea*	GH: DE Boufford 42684		KP965833/KP965858
*Potentilla tetrandra*	V: Marr & Hebda 207154		KP965834/KP965859
*Sibbaldia procumbens* s.l.	V: Hebda & Banner 198202	Haplotype A/Haplotype 1	KP965810/KP965835
*Sibbaldia procumbens* s.l.	V: BA Bennett 193317	Haplotype B/Haplotype 2	KP965811/KP965836
*Sibbaldia procumbens* s.l.	V: KL Marr 203973	Haplotype C/Haplotype 3	KP965812/KP965837
*Sibbaldia procumbens* s.l.	V: KL Marr 201839	Haplotype D/Haplotype 4	KP965813/KP965838
*Sibbaldia procumbens* s.l.	V: HJ Guest 199750	Haplotype E/Haplotype 5	KP965814/KP965839
*Sibbaldia procumbens* s.l.	V: KL Marr 191849	Haplotype F/Haplotype 6	KP965815/KP965840
*Sibbaldia procumbens* s.l.	V: W Miles 195591	Haplotype G/Haplotype 7	KP965816/KP965841
*Sibbaldia procumbens* s.l.	COLO: T Hogan 453505	Haplotype H/Haplotype 8	KP965817/KP965842
*Sibbaldia procumbens* s.l.	COLO: V Komarkova 274380	Haplotype I/Haplotype 9	KP965818/KP965843
*Sibbaldia procumbens* s.l.	V: KL Marr 203126	Haplotype J/Haplotype 10	KP965819/KP965844
*Sibbaldia procumbens* s.l.	V: KL Marr 189209	Haplotype K/Haplotype 11	KP965820/KP965845
*Sibbaldia procumbens* s.l.	UVIC: GA Allen 1328	Haplotype L/Haplotype 12	KP965821/KP965846
*Sibbaldia procumbens* s.l.	UVIC: GA Allen 1332	Haplotype M/Haplotype 13	KP965822/KP965847
*Sibbaldia procumbens* s.l.	V: HJ Guest 199762	Haplotype N/Haplotype 14	KP965823/KP965848
*Sibbaldia procumbens* s.l.	V: KL Marr 207181	Haplotype O/Haplotype 15	KP965824/KP965849
*Sibbaldia procumbens* s.l.	V: Schoenswetter 208220	Haplotype P/Haplotype 16	KP965825/KP965850
*Sibbaldia procumbens* s.l.	V: Alsos & Tribsch 192000	Haplotype Q/Haplotype 17	KP965826/KP965851
*Sibbaldia procumbens* s.l.	V: Schwienbacher 208219	Haplotype R/Haplotype 18	KP965827/KP965852
*Sibbaldia procumbens* s.l.	S: Lundberg 4	Haplotype T/Haplotype 19	KP965828/KP965853
*Sibbaldia procumbens* s.l.	V:B Leamy 25146	Haplotype U/Haplotype 20	KP965829/KP965854
*Sibbaldia procumbens* s.l.	GH: DE Boufford 36587	Haplotype V/Haplotype 21	KP965830/KP965855
*Sibbaldia procumbens* s.l.	GH: DE Boufford 38079	Haplotype W/Haplotype 22	KP965831/KP965856
*Sibbaldia procumbens* s.l.	Feng 38 (HIB)	Haplotype 23	MK085917/MK085936
*Sibbaldia procumbens* s.l.	Feng 65 (HIB)	Haplotype 24	MK085918/MK085937
*Sibbaldia procumbens* s.l.	Feng 79 (HIB)	Haplotype 25	MK085919/MK085938
*Sibbaldia procumbens* s.l.	Feng 79 (HIB)	Haplotype 26	MK085920/MK085939
*Sibbaldia procumbens* s.l.	Feng 59 (HIB)	Haplotype 27	MK085921/MK085940
*Sibbaldia procumbens* s.l.	Feng 49 (HIB)	Haplotype 28	MK085922/MK085941
*Sibbaldia procumbens* s.l.	Feng 49 (HIB)	Haplotype 29	MK085923/MK085942
*Sibbaldia procumbens* s.l.	Feng 92 (HIB)	Haplotype 30	MK085924/MK085943
*Sibbaldia procumbens* s.l.	Feng 99 (HIB)	Haplotype 31	MK085925/MK085944
*Sibbaldia procumbens* s.l.	Feng 33 (HIB)	Haplotype 32	MK085926/MK085945
*Sibbaldia procumbens* s.l.	Feng 24 (HIB)	Haplotype 33	MK085927/MK085946
*Sibbaldia procumbens* s.l.	Feng 28 (HIB)	Haplotype 34	MK085928/MK085947
*Sibbaldia procumbens* s.l.	Feng 95 (HIB)	Haplotype 35	MK085929/MK085948
*Sibbaldia procumbens* s.l.		Haplotype 36	MK085930/MK085949
*Sibbaldia procumbens* s.l.	Feng 131 (HIB)	Haplotype 37	MK085931/MK085950
*Sibbaldia procumbens* s.l.	Feng YA (HIB)	Haplotype 38	MK085932/MK085951
*Sibbaldia procumbens* s.l.	Feng YA (HIB)	Haplotype 39	MK085933/MK085952
*Sibbaldia procumbens* s.l.	F03513 (KUMA)	Haplotype 40	MK085934/MK085953
*Sibbaldia procumbens* s.l.	SHER0122 (ALTB)	Haplotype 41	MK085935/MK085954

*HIB, Herbarium of Wuhan Botanical Garden, Chinese Academy of Sciences; KUMA, Herbarium of Kumamoto University; ALTB, South Siberian Botanical Garden of Altai State University.*

According to previous studies (Dobes and Paule, 2010; Eriksson et al., 2015), *Potentilla tetrandra* (Bunge) Hook. f., *P. purpurea* (Royle) Hook. f., and *Sibbaldianthe adpressa* (Bunge) Juz were selected as outgroups and sequences were downloaded from NCBI (Table 1). The chloroplast *atpI*-*atpH* spacer, the *trnL* intron and *trnL*-*trnF* spacer were amplified from the 156 *S. procumbens* s.l. individuals using previously published primers. The primers from Shaw et al. (2007) were employed to amplify *atpI*-*atpH*; the primers (c and f) from Taberlet et al. (1991) were used to amplify *trnL*-*trnF* + *trnL* intron. The sequences (*atpI*-*atpH* spacer, the *trnL* intron and *trnL*-*trnF* spacer regions) of the 176 *S. procumbens* s.l. individuals from Allen et al. (2015) were also included in our analyses (Table 2).

**Table 2:** Sample locations, size, and haplotype frequencies.

Population code	Location	Longitude and latitude	Haplotype (haplotype frequency)
POP1	Alaska (excl. Southeast Alaska)		H2 (19), H3 (1)
POP2	Yukon		H2 (13), H4 (1)
POP3	Northwest Territories		H2 (2)
POP4	Northern Columbia and Southeast Alaska		H1 (3), H2(17), H3 (5), H4 (5), H10 (10)
POP5	Southern Columbia and Alberta		H1 (1), H2 (11), H3 (14), H4 (5), H5 (1), H10 (7)
POP6	Western United States		H1 (8), H2 (4), H3 (1), H5 (2), H6 (1), H7 (2), H8 (1), H9 (1), H10 (9), H11 (1), H12 (1), H13 (1), H14 (2)
POP7	Greenland	N69°53′22′′W53°30′14′′	H2 (1)
POP8	Svalbard, Norway	N79°23′09′′E13°26′24′′	H2 (2)
POP9	Jan Mayen island, Norway	N70°59′00′′E08°32′00′′	H2 (1)
POP10	Kulusuk, Greenland	N65°34′31′′W37°10′59′′	H2 (1)
POP11	Faeroe Islands, Denmark	N62°07′27′′W06°07′27′′	H2 (1)
POP12	Finse Ulvik, Noway	N60°36′07′′E7°30′14′′	H17 (1)
POP13	Italy	N46°24′22′′E08°04′20′′	H16 (5), H18 (1), H36 (1)
POP14	Onundarfjordur, Iceland	N66°03′00′′W23°35′00′′	H17 (1)
POP15	Skaftafell, Iceland	N64°00′58′′W16°58′19′′	H2 (1)
POP16	Malselv, Norway	N68°48′00′′E19°02′00′′	H17 (1)
POP17	Jilong, Tibet, China	N28°32′54′′E85°14′24′′	H19 (12)
POP18	Nielamu, Tibet, China	N28°14′46′′E86°00′40′′	H19 (1), H20 (3)
POP19	Dingjie, Tibet, China	N28°08′08′′E87°41′57′′	H24 (2), H25 (1), H26 (1)
POP20	Yadong, Tibet, China	N27°37′2′′E89°02′30′′	H19 (4)
POP21	Gongbujiangda, Tibet, China	N29°49′4′′E92°22′30′′	H21 (4)
POP22	Bomi, Tibet, China	N29°50′16′′E95°29′57′′	H19 (3), H27 (1)
POP23	Milin, Tibet, China	N29°29′21′′E94°55′43′′	H20 (2), H28 (1), H29 (1)
POP24	Dingqing, Tibet, China	N31°33′50′′E95°35′18′′	H21 (3), H30 (1)
POP25	Basu, Tibet, China	N30°09′14′′E97°18′42′′	H21 (7), H24 (5)
POP26	Mangkang, Tibet, China	N29°14′01′′E98°41′01′′	H23 (3), H31 (2)
POP27	Xianggelila, Yunnan, China	N27°47′10′′E99°36′44′′	H22 (1), H23 (3)
POP28	Dege, Sichuan, China	N31°06′14′′E96°30′24′′	H21 (2), H23 (1), H24 (2)
POP29	Litang, Sichuan, China	N30°17′13′′E99°34′16′′	H21 (1), H24 (4)
POP30	Jiulong, Sichuan, China	N29°21′43′′E101°29′51′′	H21 (3), H22 (7), H23(2)
POP31	Xiaojin, Sichuan, China	N30°54′55′′E102°53′26′′	H21 (1), H22 (2), H23 (3), H32 (1)
POP32	Daofu, Sichuan, China	N31°01′10′′E101°14′00′′	H21 (5), H33 (1)
POP33	Rangtang, Sichuan, China	N32°26′14′′E100°49′26′′	H21 (2), H34 (3)
POP34	Aba, Sichuan, China	N33°07′30′′E102°21′20′′	H21 (4)
POP35	Qilian, Qinghai, China	N38°07′36′′E100°13′42′′	H19 (1), H35 (1)
POP36	Meixian, Shanxi, China	N33°59′30′′E107°32′25′′	H19 (12)
POP37	Lintan, Gansu, China	N34°43′48′′E103°18′36′′	H21 (3), H34 (1)
POP38	Changbai mountain, Jilin, China	N41°51′49′′E127°52′55′′	H37 (4)
POP39	Taiwan, China	N24°08′20′′E121°16′19′′	H19 (21)
POP40	Armenia	N40°10′E44°31′	H38 (6), H39 (3)
POP41	Honshu, Japan	N35°29′47′′E138°10′1′′	H40 (1)
POP42	Russia, Komi ASSR	N60°39′18′′E65°21′04′′	H17 (1)
POP43	Russia, Altai Republic	N51°03′30′′E85°41′30′′	H15 (3), H41 (1)
POP44	Tajikistan	N38°24′00′′E73°28′48′′	H20 (1)
POP45	Georgia	N42°19′12′′E43°21′36′′	H19 (1)
Total		45 populations 332 individuals	

Polymerase chain reactions (PCR) were performed in 50 μl volumes with 10 ng of DNA template, 1 μl of each primer and 44 μl 1.1× PCR Master mix of QINGKE kit multiplex PCR (Qingke, Beijing, China). The PCR protocol included (1) 2 min at 98°C, (2) 30 cycles of 94°C for 30 s, (3) 52°C/50°C for 30 s, (4) 72°C for 30 s, and (5) 72°C for 7 min. PCR products were checked for length and concentrations on 2% agarose gels, and successfully amplified products were sent to Beijing Genomics Institute (Wuhan, Hubei, China) for Sanger sequencing. All newly produced haplotype sequences have been deposited in GenBank (Table 1).

### Phylogeographical Analyses

The sequences of each DNA region were aligned using Bioedit (Hall, 1999). The three DNA regions (*atpI*-*atpH* spacer, the *trnL* intron, and *trnL*-*trnF* spacer) were concatenated into a single combined sequence for each individual prior to analysis.

The number of haplotypes, haplotype diversity (Grismer et al., 2013), and nucleotide diversity (π) were evaluated with the software DNASP 5.10 (Librado and Rozas, 2009). The distribution of each haplotype was plotted on a map made by ArcGis 10.1 (ESRI, 2012). PERMUT 1.0 (Pons and Petit, 1996) was used to detect the average gene diversity within populations (*h*_S_), total gene diversity (Janssens et al., 2016), and the coefficients of differentiation (*G*_ST_, a population differentiation estimate based solely on haplotype frequencies, and *N*_ST_, a parameter that considers both haplotype frequencies and their genetic divergence) with 1,000 random permutations. When *N*_ST_ is significantly larger than *G*_ST_, strong phylogeographic structure is indicated. SAMOVA1.0 (Dupanloup et al., 2002) was used to identify geographically and genetically distinguishable groups. The analysis of molecular variance (AMOVA) was conducted in Arlequin 3.5 (Excoffier and Lischer, 2010).

An unrooted haplotype network was constructed using NETWORK 4.6.11 (Bandelt et al., 1999). Partitioned Bayesian analysis was carried out with MrBayes 3.1.2 (Huelsenbeck and Ronquist, 2001), under the general time reversible substitution model. The best partition scheme and substitution model was determined by PartitionFinder 1.1.1 (Lanfear et al., 2012). We executed one cold and three incrementally heated Monte Carlo Markov chains on two simultaneous runs, sampling every 1,000th generation, with a burn-in of 25%. Indels were included as (0, 1) characters in a separate data partition with software SeqState (Müller, 2005). The divergence time of haplotypes was estimated using BEAST 1.7.5 (Drummond and Rambaut, 2007), following the descriptions by Allen et al. (2015).

### Biogeographic Reconstruction

For biogeographic reconstruction, individuals of *S. procumbens* s.l. were assigned to five areas (labeled A–E) based on their present distribution: Eastern Asia, Central Asia, Western Eurasia, eastern North America, or western North America. Ancestral distributions of *S. procumbens* s.l. were inferred by BioGeoBEARS (Matzke, 2013, 2014) as implemented in RASP 4.0 (Yu et al., 2015) using 10,000 randomly sampled trees from the posterior distribution generated in the BEAST analysis, all other parameters were set to their defaults.

### Ecological Niche Modeling

Ecological niche modeling was carried out in Maxent 3.3.3k (Phillips and Dudík, 2008) to reconstruct potential geographic distribution of *S. procumbens* s.l. in different historical periods. A total of 225 records (Supplementary Table S1) spread across the distribution range were used for the analysis. Nineteen bioclimatic variables with the 2.5 arc-min spatial resolution were downloaded from WorldClim^1^, and the variables with | Pearson’s *R*|≥ 0.7 were excluded; 10 variables (BIO1, annual mean temperature; BIO2, mean diurnal range; BIO5, max temperature of warmest month; BIO7, temperature annual range; BIO8, mean temperature of wettest quarter; BIO9, mean temperature of driest quarter; BIO12, annual precipitation; BIO15, precipitation seasonality; BIO17, precipitation of driest quarter; BIO18, precipitation of warmest quarter) were selected to simulate the past, current, and future suitable areas of *S. procumbens* s.l. Model validation was performed using Maxent default settings with 10 independent replicates of subsamples. The present distribution of *S. procumbens* s.l. was established using the bioclimatic variables for the current climate (1960–1990). Future climate scenario data for 2070 (2061–2080) were obtained from global climate models (GCMs), with future climate projections based on IPCC 5th assessment data, with calibration and statistically down-scaling using the data for the “current” climate. The community climate system model 4 (CCSM4) and NCAR-CCSM were chosen for the last glacial maximum (LGM, 0.021 Ma) and the last interglacial (LIG, 0.12–0.14 Ma), respectively.

## Results

### Sequence Variation and Haplotype Distribution

The total aligned sequence length including all outgroups was 1,712 bp (base pairs). While the aligned sequence of *S. procumbens* s.l. was 1,563 bp (662 bp for the *atpI*-*atpH* spacer, 901 bp for the combined *trnL* intron and *trnL*-*trnF* spacer), with 66 variable characters (including 17 single nucleotide polymorphisms and 14 indels) (Supplementary Table S2) and 45 characters parsimony-informative characters.

**FIGURE 1:**
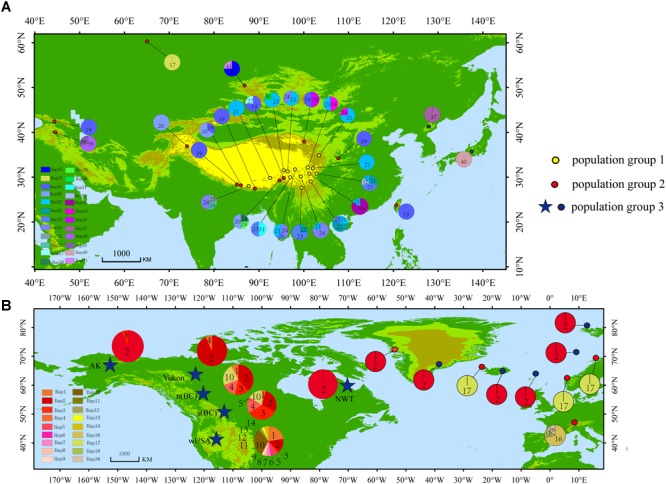
Distribution of haplotypes. **(A)** Haplotype distribution in Asia. **(B)** Haplotype distribution in North America and Europe [Modified according to Allen et al. (2015)].

A total of 41 haplotypes were detected, including 19 (Haplotypes 23–41) newly identified haplotypes in this study and 22 previously identified haplotypes by Allen et al. (2015). Haplotypes 1–18 correspond to haplotype A–R in Allen et al. (2015); haplotypes 19–22 correspond to haplotype T, U, V and W in Allen et al. (2015). Among the 28 haplotypes in Eurasia, 16 were found only in a single population, two haplotypes were found in two populations, and five were found in more than two populations (Table 2 and Figure 1). The most frequent and widespread haplotype (H19) was found in 30% of individuals and 17.6% of populations, which was widely distributed in the QTP and adjacent regions of Taiwan and Georgia. Fourteen populations contained only one haplotype (Table 2 and Figure 1). Twelve populations contained two haplotypes, six populations contained three haplotypes, and one population contained four haplotypes (Table 2 and Figure 1).

### Genetic Diversity and Structure

**FIGURE 2:**
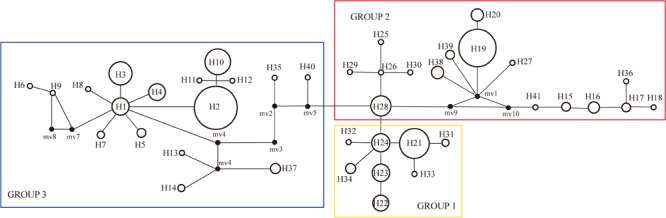
Unrooted haplotype network with SAMOVA defined groups, the size of the circles represents the frequency of haplotypes, with larger circles representing haplotypes found more frequently than smaller circles.

The haplotype diversity (Janssens et al., 2016) and the nucleotide diversity (π) was 0.8977 and 0.6 × 10^-2^, respectively, for all populations in the Northern Hemisphere. The haplotype diversity and nucleotide diversity for populations in Eurasia was 0.8394 and 0.46 × 10^-2^, respectively. For the populations in Eurasia, *h*_S_ and *h*_T_ were 0.426 and 0.871. The coefficients of differentiation measured over the 39 populations in Eurasia were *G*_ST_ = 0.515, *N*_ST_ = 0.826; and the Permutation test revealed that *N*_ST_ was significantly higher than *G*_ST_ (*P* < 0.01), which indicates phylogeographic structure for Eurasian populations. AMOVA results showed that a large proportion (92.97%) of the chloroplast variation was among populations (Table 3). The SAMOVA analysis identified three population groups (Figure 1, 2).

**Table 3:** Structure of genetic variation within and among three SAMOVA-derived groups.

Source of variation	Percentage of variation	F-statistic
**Across all population groups**		
Among groups	78.85	
Among populations within groups	13.06	*FCT* = 0.78846^∗∗∗^
Within populations	8.1	*FSC* = 0.61722^∗∗∗^
Total		*FST* = 0.91903^∗∗∗^
**Group 1 vs. Group 2**		
Among groups	74.02	
Among populations within groups	19.4	*FCT* = 0.74018^∗∗∗^
Within populations	6.59	*FSC* = 0.74651^∗∗∗^
Total		*FST* = 0.93414^∗∗∗^
**Group 1 vs. Group 3**		
Among groups	88.34	
Among populations within groups	2.48	*FCT* = 0.88335^∗∗∗^
Within populations	9.18	*FSC* = 0.21285^∗∗∗^
Total		*FST* = 0.90818^∗∗∗^
**Group 2 vs. Group 3**		
Among groups	75.99	
Among populations within groups	15.68	*FCT* = 0.75988^∗∗∗^
Within populations	8.33	*FSC* = 0.65293^∗∗∗^
Total		*FST* = 0.91666^∗∗∗^

*^∗∗∗^*P* < 0.0001; *FCT*, differentiation among groups within the species; *FSC*, differentiation among populations within groups; *FST*, differentiation among populations within the species.*

**FIGURE 3:**
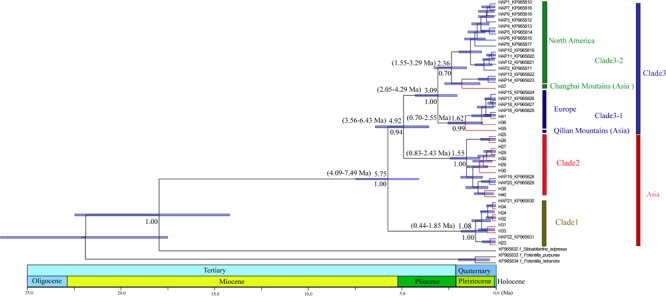
Phylogenetic relationships among haplotypes. Numbers below branches denote posterior probability. Number above nodes indicates the estimated time to the most recent common ancestor. The haplotypes with blue color branches represent the haplotypes from Allen et al. (2015).

PartitionFinder determined one partition (*trnL*-*trnF* + *trnL* intron + *atpI-atpH*) as the best scheme. The phylogenetic analysis of all haplotypes revealed three clades (Figure 3). The early diverging clade (clade 1) includes eight haplotypes, H21–H24 and H31–H34, which are all endemic to the QTP and adjacent regions. Clade 2 consists of 11 haplotypes (H15–H20, H25–H30, H36–H41), which are endemic to Asia with a wider current distribution range than clade 1. The haplotypes in clade 3 form two small subclades; the first subclade consists of 15 haplotypes (H1–H14, H37), with H1 and H3–H14 distributed in North America, with H2 having a wide distribution around North America and extending east to Northwest Europe. H37 is distributed in the Changbai Mountains in the northeast of China; the second subclade includes seven haplotypes, which are mostly distributed in Europe (H15–H18, H36, H41), with only H35 distributed in the Qilian Mountains (north of the QTP) in China.

The divergence of *S. procumbens* s.l. was estimated to be 5.75 Ma (95% HPD 4.09–7.49 Ma); the split between clade 2 and clade 3 was dated to be around 4.92 Ma (95% HPD 3.56–6.43 Ma); subsequent divergence within clades 2 and 3 was around 1.55 Ma (95% HPD 0.83–2.43 Ma) and 3.09 Ma (95% HPD 2.05–4.29 Ma), respectively.

**FIGURE 4:**
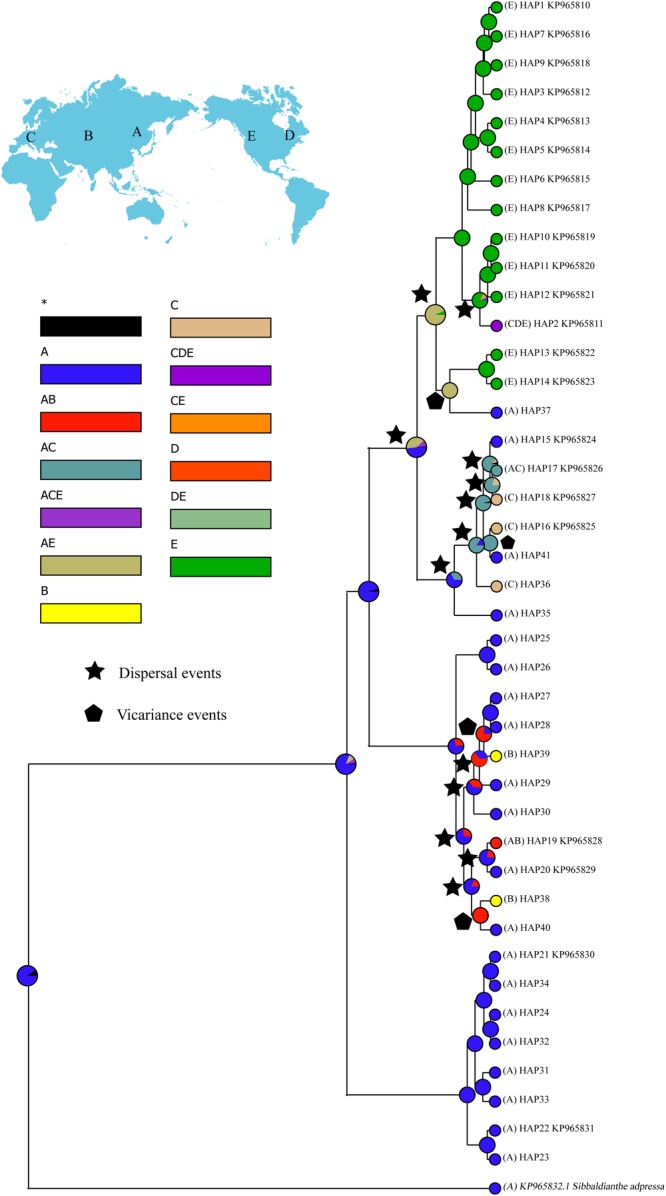
Ancestral distributions reconstructed by RASP. A, Eastern Asia; B, Central Asia; C, Western Eurasia; D, eastern North America; E, western North America.

RASP analysis resolved Eastern Asia as the most likely ancestral region of *S. procumbens* s.l. (Figure 4). Fifteen dispersal and four vicariance events were identified.

### Past, Present, and Future Ecological Niche Models

**FIGURE 5:**
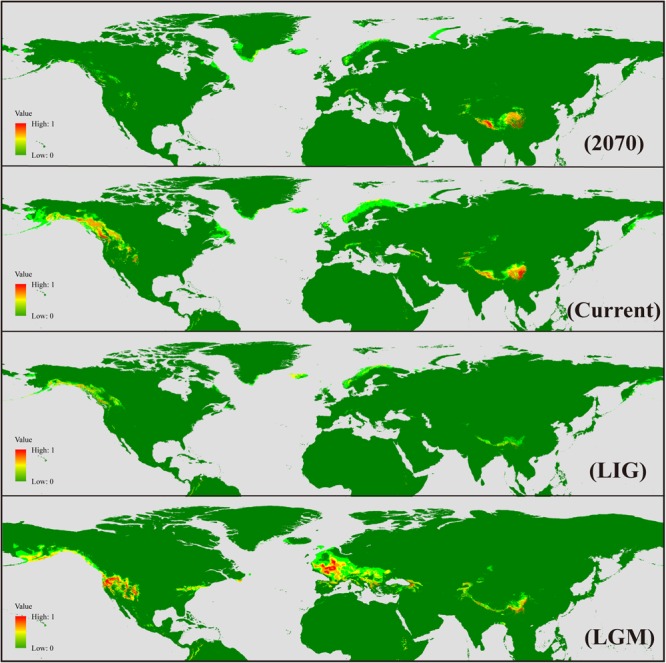
Geographical distribution models showing climatic suitability for *Sibbaldia procumbens* s.l. for 2070, present time, last interglacial (LIG), and last glacial maximum (LGM).

The AUC (area under curve) value for the potential distribution of *S. procumbens* s.l. was high (0.978 for future, 0.979 for LGM, and 0.95 for LIG), indicating good predictive model performance. The predicted distribution under current conditions was generally similar to its observed distribution across the Northern Hemisphere, with western North America and the QTP as the main distribution areas, and sparse distribution in Greenland, the Mediterranean, and Taiwan. At the LGM, the range of *S. procumbens* s.l. showed an expansion in distribution to the Caucasus Mountains and north of the Mediterranean (the Alps and Scandinavian Peninsula); the expansion was also detected in the Western Rocky Mountains and the Eastern Appalachian Mountains (Figure 5). At the LIG, the range of *S. procumbens* s.l. shrunk to the tallest mountain peaks in the QTP, western North America, and Scandinavia (Figure 5). The predicted distribution of *S. procumbens* s.l. in the future (2061–2080) is contracted, mainly concentrated in the QTP and adjacent regions (Figure 5).

## Discussion

### Haplotype Divergence During Neogeneand Quaternary

Climate change and tectonic motion are thought as important factors driving species formation and differentiation (Bruch et al., 2006; Fortelius et al., 2006; Johnson et al., 2006). According to our estimation, the diversification of *S. procumbens* s.l. is likely to have begun around 5.75 Ma (95% HPD 4.09–7.49 Ma) in the late Miocene (Figure 3). A global second cooling happened 11.6–5.3 Ma (Molnar et al., 2010), which could have driven the initial divergence of *S. procumbens* s.l. In addition, the early diverging haplotypes (clade 1 in Figure 3) of *S. procumbens* s.l. are distributed in the QTP (Figure 1), and the fifth stage uplift of the Himalayas occurred about 7–5 Ma (Harrison et al., 1992; Yin, 2006), which may have been another factor driving the initial diversification within *S. procumbens* s.l.

The divergence between the Asia clade (clade 2) and the North America + Europe clade (clade 3) was estimated to be around the early Pliocene (Figure 3). This divergence likely resulted from the spread of *S. procumbens* s.l. from the QTP to other regions. A series of mountain ranges extending from the Himalayas are thought to have facilitated the spread and diversification of this species (Allen et al., 2015).

The migration of *S. procumbens* s.l. from Asia into North America via the Bering Strait was estimated to be around 2.36 Ma (95% HDP 1.55–3.29 Ma), which generally coincides with the estimation by Allen et al. (2015). The reduction of the sea level between Asia and Alaska during the late Pliocene may have facilitated the spread of many species (Vermeij, 1991; Berggren, 1995). Long-distance dispersal could also play an important role in the distribution expansion of *S. procumbens* s.l. (Givnish et al., 2004; Nathan, 2006; Mummenhoff and Franzke, 2007); since the seeds are small achenes (Coker, 1966; Li et al., 2003) which can be transmitted with wind or germinate after passage through the digestive tract of birds and other herbivores (Myers et al., 2004; Nathan, 2006; Mummenhoff and Franzke, 2007).

Quaternary glacial–interglacial cycles played important roles in the formation and differentiation of species (Nie et al., 2005; Meng et al., 2007; Yang et al., 2008; Wang et al., 2009). Periodic climatic fluctuations in the Quaternary were considered one of the main factors that formed geographical patterns of modern alpine plants (Yang et al., 2008; Wang et al., 2009; Zhang et al., 2014). In the current study, we found the greatest differentiation of *S. procumbens* s.l. occurred during the Quaternary (Figure 3), which indicate the glacial–interglacial cycles are likely factors contributing to the recent diversification within this species.

In general, our data suggest that it is likely the rapid landmass uplifts of the QTP and the global cooling during the Neogene that promoted the speciation and spread of *S. procumbens* s.l., while the Quaternary glacial–interglacial cycles have driven the recent differentiation of this species.

### Spread of *Sibbaldia procumbens* s.l.

Even though the fossil evidence of *S. procumbens* s.l. is rare, our phylogenetic analysis (Figure 3) resolved an early diverging clade composed of eight haplotypes which were all distributed in the QTP and adjoining regions suggesting the origin of the species occurred in the QTP or adjoining regions. This corresponds with the results of RASP analysis (Figure 4), which indicate Eastern Asia as the most likely ancestral region of *S. procumbens* s.l. The QTP and adjoining regions is one of the world’s most important centers of biodiversity due to its high species richness and abundance of endemic species (Myers et al., 2000; Luo et al., 2016). This region has been suggested as an important origin and differentiation area for taxa with intercontinental disjunct distribution (Nie et al., 2005; Wang et al., 2007; Zhang et al., 2014).

Birkeland (2012) and Allen et al. (2015) proposed *S. procumbens* s.l. migrated both eastward and westward from Asia. The distribution patterns and phylogenetic analysis of haplotypes (Figure 1–3), and a series of dispersal events identified by the RASP analysis (Figure 4) support this proposal. More specifically, *S. procumbens* s.l. likely originated from the QTP and adjacent regions in the uplift phase during the late Miocene. The global cooling during this period provided a wider habitat, likely promoting the spread of this species, and the high mountain ranges extending from the QTP provided corridors for the spread outward: (1) to the west, *S. procumbens* s.l. dispersed along the Himalayas to the West Pamir Mountains (Tajikistan), across the northern Iranian Plateau to the Caucasus, continued to spread westward to the Balkan and Carpathian Mountains, then along the Alpine Mountains to the Scandinavian Mountains, finally arriving to lands and islands in the North Atlantic Ocean, and even to eastern North America and (2) to the east, the species likely first spread to the mountain ranges north of QTP (e.g., the Qilian Mountains and Kunlun Mountains), and moved further along the mountain range to the Tian Shan and Altai Mountains, then extended south and east to Siberia, finally reaching western North America via the Bering Strait. The North American clade was further divided into two subclades (Figure 3), the first is widespread and presents high haplotype diversity and the second consists of individuals in California and the Changbai Mountains. This supports the hypothesis of Allen et al. (2015) that *S. procumbens* s.l. shifted far southward after arriving to North America.

Additionally, *S. procumbens* s.l. could have also spread along the Qilian Mountains, Helan Mountains, Yin Mountains, and Yan Mountains to the Changbai Mountains in the northeast of China, and later arriving to Japan. A series of mountains (e.g., Qinling Mountains, Daba Mountains, Dabie Mountains, and Wuyi Mountains) could have been the route for the spread of *S. procumbens* s.l. from the QTP to Taiwan. Currently we cannot rule out the possibility that *S. procumbens* s.l. found in Taiwan are from Japan, or vice versa.

### Refugia in Eastern Asia

Refugia are geographical areas of sheltered topography that provided suitable stable microclimates allowing species to persist throughout climatic oscillations (Rowe et al., 2004; Provan and Bennett, 2008). The refugia can be identified from fossils, paleo environmental data (Comes and Kadereit, 1998; Petit et al., 2002), and phylogeographic information (Stehlik, 2003; Tribsch and Schönswetter, 2003; Provan and Bennett, 2008; Eidesen et al., 2013). Multiple Pleistocene refugia for *S. procumbens* s.l. in North America and Europe have been reported (California and the Southern Rocky Mountains; Eidesen et al., 2013; Allen et al., 2015). Similarly, several refugia in Asia for *S. procumbens* s.l. during the LGM have been suggested in the current study.

As indicated by Allen et al. (2015) and Eidesen et al. (2007), genetic differences between populations in different regions suggest long separation through glacial intervals. In Asia, scattered distribution of *S. procumbens* s.l. are found in the Changbai Mountains in the northeast of China and in Japan. Both regions possess unique private haplotypes, implying both regions have been geographically separated for one or more glacial cycles, and could have served as refugia for *S. procumbens* s.l. during the LGM.

High haplotype diversity was discovered in multiple populations (Bomi, Dingqing, Mangkang, Xiaojin, and Daofu) in the eastern QTP (Table 1 and Figure 1, 4), implying micro-refugia may have existed along the mountains. We hypothesize *S. procumbens* s.l. originated in the eastern QTP and then colonized other mountain ranges surrounding the QTP. The mountains with high elevation provided the conditions necessary for differentiation of the species. There is only one haplotype (Haplotype 19) in the island of Taiwan; while haplotype 19 is also widespread across Asia. This implies Taiwan may have been recolonized by *S. procumbens* s.l. during interglacial periods, rather than serving as one refugium for *S. procumbens* s.l. during the LGM.

## Conclusion

Our study implies current geographic and genetic distribution of *S. procumbens* s.l. is likely to have been shaped by changing climates in both the Neogene and Quaternary periods. Multiple regions in Eastern Asia may have served as important refugia for *S. procumbens* s.l. during extreme climatic events. Long-distance dispersal and vicariance may have played an important role in shaping extant distribution patterns of *S. procumbens* s.l. The distribution range of *S. procumbens* s.l. appeared to have experienced expansion and contraction during the LGM and LIG, respectively; in the future, when the global climate becomes warmer with rising carbon dioxide, the distribution of *S. procumbens* s.l. will shrink and be limited to the QTP and adjacent regions.

## Data Availability

The datasets generated for this study can be found in National Center for Biotechnology Information, Please see Table 1 in the manuscript.

## Author Contributions

Y-XS, H-CW, and HS conceived and designed this study. H-JZ, TF, and XZ performed the experiments and analyzed the data. H-JZ, JL, and Y-XS wrote the manuscript. TD, A-PM, and TF aided in filed collections. All authors read and approved the final manuscript.

## Conflict of Interest Statement

The authors declare that the research was conducted in the absence of any commercial or financial relationships that could be construed as a potential conflict of interest.

## Footnotes

1 http://www.worldclim.org/
