# Supplementary material for: Molecular Phylogeography and Ecological Niche Modeling of Sibbaldia procumbens s.l. (Rosaceae)
Source: Front Genet. 2019 Mar 13;10:201. doi: 10.3389/fgene.2019.00201 (PMC6424895; doi:10.3389/fgene.2019.00201)
Supplement: Supplementary file 1 [file Table_1.doc]

Table S1. Sample distribution information for ecological niche modelling. A total of 53 records are presented here, another 172 records can be found from Appendix S1 in Allen et al. (2015). HIB, Herbarium of Wuhan Botanical Garden; ALTB, South Siberian Botanical Garden of Altai State University; TAI, Herbarium of National Taiwan University; KUMA, Herbarium of Kumamoto University.

| Taxon | Specimen voucher | COUNTRY & LOCALITY | Latitude | Longtitude | Collector & Year | Voucher location |
| --- | --- | --- | --- | --- | --- | --- |
| *S. procumbens* s.l. | Feng 49 | Mi-Lin, Tibet, China | 29°29′21″N | 094°55′43″E | Tao Feng (2011) | HIB |
| *S. procumbens* s.l. | Feng 56 | Lin-Zhi, Tibet, China | 29°36′47″N | 094°39′32″E | Tao Feng (2011) | HIB |
| *S. procumbens* s.l. | Feng 59 | Bo-Mi, Tibet, China | 29°50′16″N | 095°29′57″E | Tao Feng (2011) | HIB |
| *S. procumbens* s.l. | Feng 61 | Bo-Mi, Tibet, China | 29°45′50″N | 095°41′13″E | Tao Feng (2011) | HIB |
| *S. procumbens* s.l. | Feng 63 | Cha-Yu, Tibet, China | 29°18′44″N | 097°00′58″E | Tao Feng (2011) | HIB |
| *S. procumbens* s.l. | Feng 65 | Ba-Su, Tibet, China | 30°09′14″N | 097°18′42″E | Tao Feng (2011) | HIB |
| *S. procumbens* s.l. | Feng 66 | Bo-Mi, Tibet, China | 29°44′24″N | 095°42′15″E | Tao Feng (2011) | HIB |
| *S. procumbens* s.l. | Feng 70 | Gong-bu-jiang-da, Tibet, China | 29°49′04″N | 092°22′30″E | Tao Feng (2011) | HIB |
| *S. procumbens* s.l. | Feng 75 | Ya-Dong, Tibet, China | 27°37′02″N | 089°02′30″E | Tao Feng (2011) | HIB |
| *S. procumbens* s.l. | Feng 78 | Ding-Jie, Tibet, China | 28°08′08″N | 087°41′57″E | Tao Feng (2011) | HIB |
| *S. procumbens* s.l. | Feng 79 | Ding-Jie, Tibet, China | 27°53′51″N | 087°33′15″E | Tao Feng (2011) | HIB |
| *S. procumbens* s.l. | Feng 84 | Nie-la-mu, Tibet, China | 28°14′46″N | 086°00′40″E | Tao Feng (2011) | HIB |
| *S. procumbens* s.l. | Feng 85 | Nie-la-mu, Tibet, China | 28°05′26″N | 085°59′57″E | Tao Feng (2011) | HIB |
| *S. procumbens* s.l. | Feng 87 | Ji-Long, Tibet, China | 28°32′54″N | 085°14′24″E | Tao Feng (2011) | HIB |
| *S. procumbens* s.l. | Feng 90 | Lei-wu-qi, Tibet, China | 31°06′14″N | 096°30′24″E | Tao Feng (2011) | HIB |
| *S. procumbens* s.l. | Feng 92 | Ding-Qing, Tibet, China | 31°33′50″N | 095°35′18″E | Tao Feng (2011) | HIB |
| *S. procumbens* s.l. | Feng 73 | Ya-Ma, Tibet, China | 27°45′37″N | 089°08′59″E | Tao Feng (2011) | HIB |
| *S. procumbens* s.l. | Feng 95 | Qi-Lian, Qing-hai, China | 38°07′36″N | 100°13′42″E | Tao Feng (2011) | HIB |
| *S. procumbens* s.l. | Feng 11 | Xiang-ge-li-la, Yunnan, China | 27°47′10″N | 099°36′44″E | Tao Feng (2011) | HIB |
| *S. procumbens* s.l. | Feng 12 | De-Qin, Yunnan, China | 28°19′41″N | 099°06′17″E | Tao Feng (2011) | HIB |
| *S. procumbens* s.l. | Feng 99 | Mang-Kang, Tibet, China | 29°14′01″N | 098°41′01″E | Tao Feng (2011) | HIB |
| *S. procumbens* s.l. | Feng 13 | Li-Tang, Sichuan, China | 30°17′13″N | 099°34′16″E | Tao Feng (2011) | HIB |
| *S. procumbens* s.l. | Feng 15 | Xiang-Cheng, Sichuan, China | 28°40′23″N | 099°49′35″E | Tao Feng (2011) | HIB |
| *S. procumbens* s.l. | Feng 16 | Xiang-Cheng, Sichuan, China | 29°08′01″N | 100°02′58″E | Tao Feng (2011) | HIB |
| *S. procumbens* s.l. | Feng 17 | Li-Tang, Sichuan, China | 29°33′16″N | 100°17′52″E | Tao Feng (2011) | HIB |
| *S. procumbens* s.l. | Feng 18 | Li-Tang, Sichuan, China | 30°05′07″N | 100°28′52″E | Tao Feng (2011) | HIB |
| *S. procumbens* s.l. | Feng 19 | Gan-Zi, Sichuan, China | 31°44′12″N | 099°34′33″E | Tao Feng (2011) | HIB |
| *S. procumbens* s.l. | Feng 20 | Ma-ni-gan-ge, Sichuan, China | 31°52′56″N | 099°02′37″E | Tao Feng (2011) | HIB |
| *S. procumbens* s.l. | Feng 21 | De-Ge, Sichuan, China | 31°56′06″N | 098°55′46″E | Tao Feng (2011) | HIB |
| *S. procumbens* s.l. | Feng 22 | Gan-Zi, Sichuan, China | 31°36′08″N | 100°11′17″E | Tao Feng (2011) | HIB |
| *S. procumbens* s.l. | Feng 23 | Lu-Huo,Sichuan， China | 30°46′53″N | 101°18′48″E | Tao Feng (2011) | HIB |
| *S. procumbens* s.l. | Feng 24 | Dao-Fu, Sichuan, China | 31°01′10″N | 101°14′00″E | Tao Feng (2011) | HIB |
| *S. procumbens* s.l. | Feng 28 | Rang-Tang, Sichuan, China | 32°26′14″N | 100°49′26″E | Tao Feng (2011) | HIB |
| *S. procumbens* s.l. | Feng 29 | Rang-Tang, Sichuan, China | 32°51′40″N | 101°41′44″E | Tao Feng (2011) | HIB |
| *S. procumbens* s.l. | Feng 30 | A-Ba, Sichuan, China | 33°07′30″N | 102°21′20″E | Tao Feng (2011) | HIB |
| *S. procumbens* s.l. | Feng 31 | Hong-Yuan, Sichuan, China | 32°20′16″N | 102°26′45″E | Tao Feng (2011) | HIB |
| *S. procumbens* s.l. | Feng 32 | Xiao-Jin, Sichuan, China | 31°42′22″N | 102°18′49″E | Tao Feng (2011) | HIB |
| *S. procumbens* s.l. | Feng 33 | Xiao-Jin, Sichuan, China | 30°54′55″N | 102°53′26″E | Tao Feng (2011) | HIB |
| *S. procumbens* s.l. | Feng 36 | Kang-Ding, Sichuan, China | 30°04′28″N | 101°48′13″E | Tao Feng (2011) | HIB |
| *S. procumbens* s.l. | Feng 38 | Jiu-Long, Sichuan, China | 29°21′43″N | 101°29′51″E | Tao Feng (2011) | HIB |
| *S. procumbens* s.l. | Feng 41 | Mu-Li, Sichuan, China | 28°07′31″N | 101°09′36″E | Tao Feng (2011) | HIB |
| *S. procumbens* s.l. | Feng (YA) | Armenia | 39°41′12″N | 046°03′10″E | Chris J. Davis (2014) | HIB |
| *S. procumbens* s.l. | Feng 109 | Mei-Xian, Shanxi, China | 33°59′30″N | 107°32′25″E | Tao Feng (2011) | HIB |
| *S. procumbens* s.l. | Feng 115 | Lin-Tan, Gansu, China | 34°43′48″N | 103°18′36″E | Tao Feng (2011) | HIB |
| *S. procumbens* s.l. | Feng 117 | Lin-Tan, Gansu, China | 34°30′30″N | 103°20′25″E | Tao Feng (2011) | HIB |
| *S. procumbens* s.l. | Feng 131 | Chang-bai Mountain, Jilin, China | 41°51′49″N | 127°52′55″E | Tao Feng (2011) | HIB |
| *S. procumbens* s.l. | AHER 0122 | Komi ASSR, Russia | 60°30′18″N | 65°21′04″E | Chernyshov M (2014) | ALTB |
| *S. procumbens* s.l. | ASHK510 | Altai Republic | 51°05′30″N | 85°41′15″E | Larionov T (2014) | ALTB |
| *S. procumbens* s.l. | 112287 | Salzburg, Austria | 47°03′27″N | 012°59′58″E | Sonnblick (2013) | ALTB |
| *S. procumbens* s.l. | 286834 | Taiwan, China | 24°08′20″N | 121°16′19″E | Zhilong Liu (2017) | TAI |
| *S. procumbens* s.l. | 286835 | Taiwan, China | 24°09′11″N | 121°17′02″E | Zhilong Liu (2017) | TAI |
| *S. procumbens* s.l. | Zhang BM | Deqin, Yunnan, China | 28°21′34″E | 099°03′52″E | Huajie Zhang (2017) | HIB |
| *S. procumbens* s.l. | F03513 | Honshu, Japan | 35°29′47″N | 138°10′01″E | Noriyuki Fujii (2005) | KUMA |
