# Supplementary material for: Molecular Phylogeography and Ecological Niche Modeling of Sibbaldia procumbens s.l. (Rosaceae)
Source: Front Genet. 2019 Mar 13;10:201. doi: 10.3389/fgene.2019.00201 (PMC6424895; doi:10.3389/fgene.2019.00201)
Supplement: Supplementary file 2 [file Table_2.doc]

TABLE S2. Characteristics of 41 haplotype sequences. "-" means gap; E=GCCAAA; F=TAT; M=CTTTATTTATACCTTATATTTA; N=CTTTATTTATACCTTATATTTA; R=TAATTAAAAATG; S=TGTA; Q=TTAG; X=CTTTATTT; Z=ATACCTTATATTTA; Y=ATACCTTATATTTACTTTATTT.

|  | H1 | H2 | H3 | H4 | H5 | H6 | H7 | H8 | H9 | H10 | H11 | H12 | H13 | H14 | H15 | H16 | H17 | H18 | H19 | H20 | H21 | H  22 | H23 | H24 | H25 | H26 | H27 | H28 | H29 | H30 | H31 | H32 | H33 | H34 | H35 | H36 | H37 | H38 | H39 | H40 | H41 |
| --- | --- | --- | --- | --- | --- | --- | --- | --- | --- | --- | --- | --- | --- | --- | --- | --- | --- | --- | --- | --- | --- | --- | --- | --- | --- | --- | --- | --- | --- | --- | --- | --- | --- | --- | --- | --- | --- | --- | --- | --- | --- |
| 40-  45 | - | - | - | - | - | - | - | - | - | - | - | - | - | - | E | E | E | E | E | E | E | E | E | E | E | E | E | E | E | E | E | E | E | E | - | E | - | E | E | - | E |
| 77 | · | · | · | · | · | · | · | · | · | · | · | · | · | · | · | · | · | · | · | · | · | · | · | · | · | · | · | · | · | · | · | · | · | · | A | · | · | · | · | · | · |
| 102 | · | · | · | · | · | · | · | · | · | · | · | · | · | · | C | C | C | C | · | · | · | · | · | · | · | · | · | · | · | · | · | · | · | · | · | C | · | · | · | · | C |
| 132 | · | · | · | · | · | · | · | · | · | · | · | · | · | · | · | · | · | · | G | G | · | · | · | · | · | · | · | · | · | · | · | · | · | · | · | · | · | · | · | · | · |
| 137 | · | · | · | · | · | · | · | · | · | · | · | · | · | · | · | · | · | · | · | · | · | · | · | · | · | · | · | · | · | · | · | · | A | · | · | · | · | · | · | · | · |
| 141 | - | - | - | - | T | - | - | - | - | - | - | - | - | - | - | - | - | - | - | - | - | - | - | - | - | - | - | - | - | - |  | - | - | - | - | - | - | - | - | - | - |
| 233 | · | · | · | · | · | · | · | · | · | · | · | · | · | G | · | · | · | · | · | · | · | · | · | · | · | · | · | · | · | · | · | · | · | · | · | · | · | · | · | · | · |
| 260 | · | · | · | · | · | · | · | · | · | · | · | · | C | C | C | C | C | C | C | C | C | C | C | C | C | C | C | C | C | C | C | C | C | C | - | - | C | C | C | · | C |
| 266 | · | · | · | · | · | · | · | · | · | · | · | · | · | · | · | · | · | · | · | · | · | · | · | · | · | · | · | · | · | · | · | · | T | · | · | · | · | · | · | · | · |
| 285 | · | · | · | · | · | · | · | · | · | · | · | · | · | · | · | · | · | · | · | · | · | · | · | · | · | · | · | · | · | · | · | · | · | · | · | · | · | · | · | · | · |
| 335-345 | · | · | · | · | · | · | · | · | · | · | · | · | - | - | · | · | · | · | · | · | · | · | · | · | · | · | · | · | · | · | · | · | · | · | · | · | · | · | · | · | · |
| 346 | · | · | · | · | · | · | · | · | · | · | · | · | · | · | · | · | · | · | · | · | · | · | · | · | · | · | · | · | · | A | · | · | · | · | · | · | · | · | · | · | · |
| 360 | · | · | · | · | · | · | · | · | · | · | · | · | · | · | · | · | · | · | · | · | · | · | · | · | · | · | · | · | · | · | · | · | · | · | · | · | T | · | · | · | · |
| 400 | · | G | · | · | · | · | · | · | · | G | G | G | · | · | · | · | · | · | · | · | · | · | · | · | · | · | · | · | · | · | · | · | · | · | G | · | · | · | · | G | · |
| 437-441 | · | · | · | · | · | · | · | · | · | · | · | · | · | · | · | · | · | · | · | · | · | · | · | · | · | · | · | · | · | · | · | - | · | · | · | · | · | · | · | · | · |
| 442-443 | · | · | · | · | · | · | · | · | · | · | · | · | · | · | CT | CT | CT | CT | · | · | · | · | · | · | · | · | · | · | · | · | · |  | · | · | · | CT | · | · | · | · | CT |
| 444 | - | - | - | - | - | - | - | - | - | - | - | - | - | - | T | T | T | T | A | A | - | - | - | - | - | - | A | - | - | - | - | - | - | - | - | T | - | A | A | - | T |
| 445 | - | - | - | - | - | - | - | - | - | - | - | - | - | - | T | T | T | T | T | T | - | - | - | - | - | - | T | - | - | - | - | - | - | - | - | T | - | T | T | - | T |
| 446 | - | - | - | - | - | - | - | - | - | - | - | - | - | - | A | A | A | A | T | T | - | - | - | - | - | - | T | - | - | - | - | - | - | - | - | A | - | T | T | - | A |
| 447 | - | - | - | - | - | - | - | - | - | - | - | - | - | - | T | T | T | T | T | T | - | - | - | - | - | - | T | - | - | - | - | - | - | - | - | T | - | T | T | - | T |
| 448 | - | - | - | - | - | - | - | - | - | - | - | - | - | - | T | T | T | T | A | A | - | - | - | - | - | - | A | - | - | - | - | - | - | - | - | T | - | A | A | - | T |
| 449-450 | - | - | - |  | - | - | - | - | - | - | - | - | - | - | TA | TA | TA | TA | TA | TA | - | - | - | - | - | - | TA | - | - | - | - | - | - | - | - | TA | - | TA | TA | - | TA |
| 451-455 | - | - | - | - | - | - | - | - | - | - | - | - | - | - | TACCT | TACCT | TACCT | TACCT | CCTTA | CCTTA | - | - | - | - | - | - | CCTTA | - | - | - | - | - | - | - | - | TACCT | - | CCTTA | CCTTA | - | TACCT |
| 456-458 | - | - | - | - | - | - | - | - | - | - | - | - | - | - | F | F | F | F | F | F | - | - | - | - | - | - | F | - | - | - | - | - | - | - | - | F | - | - | F | - | F |
| 459 | - | - | - | - | - | - | - | - | - | - | - | - | - | - | A | A | A | A | T | T | - | - | - | - | - | - | T | - | - | - | - | - | - | - | - | A | - | T | T | - | A |
| 460 | - | - | - | - | - | - | - | - | - | - | - | - | - | - | T | T | T | T | T | T | - | - | - | - | - | - | T | - | - | - | - | - | - | - | - | T | - | T | T | - | T |
| 461 | - | - | - | - | - | - | - | - | - | - | - | - | - | - | T | T | T | T | A | A | - | - | - | - | - | - | A | - | - | - | - | - | - | - | - | T | - | A | A | - | T |
| 462 | - | - | - | - | - | - | - | - | - | - | - | - | - | - | T | T | T | T | T | T | - | - | - | - | - | - | T | - | - | - | - | - | - | - | - | T | - | T | T | - | T |
| 463 | - | - | - | - | - | - | - | - | - | - | - | - | - | - | A | A | A | A | - | - | - | - | - | - | - | - | - | - | - | - | - | - | - | - | - | A | - | - | - | - | A |
| 464-485 | - | - | - | - | - | - | - | - | - | - | - | - | - | - | - | M | M | M | - | - | - | - | - | - | - | - | - | - | - | - | - | - | - | - | - | M | - | - | - | - | - |
| 486-507 | - | - | - | - | - | - | - | - | - | - | - | - | - | - | - | - | N | N | - | - | - | - | - | - | - | - | - | - | - | - | - | - | - | - | - | N | - | - | - | - | - |
| 508-515 | - | - | - | - | - | - | - | - | - | - | - | - | - | - | - | - | - | X | - | - | - | - | - | - | - | - | - | - | - | - | - | - | - | - | - | X | - | - | - | - | - |
| 516-537 | - | - | - | - | - | - | - | - | - | - | - | - | - | - | - | - | - | Y | - | - | - | - | - | - | - | - | - | - | - | - | - | - | - | - | - | - | - | - | - | - | - |
| 538-551 | - | - | - | - | - | - | - | - | - | - | - | - | - | - | - | - | - | Z | - | - | - | - | - | - | - | - | - | - | - | - | - | - | - | - | - | Z | - | - | - | - | - |
| 552 | - | - | - | - | - | - | - | - | - | - | - | - | - | - | T | T | T | T | - | - | - | - | - | - | - | - | - | - | - | - | - | - | - | - | - | T | - | - | - | - | T |
| 553 | - | - | - | - | - | - | - | - | - | - | - | - | - | - | C | C | C | C | C | C | - | - | - | - | - | - | C | - | - | - | - | C | - | - | - | C | - | C | C | - | C |
| 577 | - | - | - | - | - | - | - | - | - | - | G | - | - | - | - | - | - | - | - | - | - | - | - | - | - | - | - | - | - | - | - | - | - | - | - | - | - | - | - | - | - |
| 606 | - | - | - | - | - | - | - | - | - | - | - | - | T | T | - | - | - | - | - | - | - | - | - | - | - | - | - | - | - | - | - | - | - | - | - | - | - | - | - | - | - |
| 703 | - | - | - | - | - | - | - | - | - | - | - | - | - | - | - | - | - | - | - | - | - | - | - | - | - | - | - | - | - | - | - | - | - | - | - | - | A | - | - | - | - |
| 764 | - | - | - | - | - | - | - | - | - | - | - | - | - | - | A | A | A | A | - | - | - | - | - | - | - | - | - | - | - | - | - | - | - | - | A | A | - | - | - | - | A |
| 808 | - | - | - | - | - | - | - | - | - | - | - | - | - | - | - | - | - | - | - | - | - | - | - | - | - | - | - | - | - | - | - | - | - | - | - | - | - | - | - | C | - |
| 833 | - | - | - | - | - | - | C | - | - | - | - | - | - | - | - | - | - | - | - | - | - | - | - | - | - | - | - | - | - | - | - | - | - | - | - | - | - | - | - | - | - |
| 862 | . | . | . | . | . | . | . | . | . | . | . | . | . | . | . | . | . | . | . | . | . | . | . | . | . | . | . | . | A | . | . | . | . | . | . | . | . | . | . | . | . |
| 889-890 | - | - | - | - | - | - | - | - | - | - | - | - | - | - | - | - | - | - | - | - | - | - | - | - | - | - | - | - | - | - | - | - | - | AT | - | - | - | - | - | - | - |
| 892 | . | . | . | . | . | . | . | . | . | . | . | . | . | . | . | . | . | . | . | . | . | . | . | . | . | . | . | . | . | . | . | . | . | . | . | . | . | A | . | A | . |
| 906 |  |  |  |  |  |  |  |  |  |  |  |  |  |  |  |  |  |  | - | - | - | - | - | - | - | - | - | - | - | - | - | - | - | - |  |  |  | - | - | - |  |
| 909 | . | . | . | . | . | . | . | . | . | . | . | . | . | . | . | . | . | . | . | . | . | . | . | . | C | C | . | . | . | . | . | . | . | . | . | . | . | . | . | . | . |
| 918 | . | . | . | . | . | . | . | . | . | . | . | . | . | . | G | G | G | G | . | . | . | . | . | . | . | . | . | . | . | . | . | . | . | . | G | G | . | . | . | . | G |
| 929 | . | . | . | . | . | . | . | . | . | . | . | . | . | . | . | . | . | . | . | . | C | C | C | C | . | . | . | . | . | . | C | C | C | C | . | . | . | . | . | . | . |
| 930 | . | . | . | . | . | . | . | . | . | . | . | A | . | . | . | . | . | . | . | . | . | . | . | . | . | . | . | . | . | . | . | . | . | . | . | . | . | . | . | . | . |
| 937 | . | . | . | . | . | A | . | . | . | . | . | . | . | . | . | . | . | . | . | . | . | . | . | . | . | . | . | . | . | . | . | . | . | . | . | . | . | . | . | . | . |
| 938-939 |  | - | - | - | - | CT | - | - | - | - | - | - | - | - | - | - | - | - | - | - | - | - | - | - | - | - | - | - | - | - | - | - | - | - | - | - | - | - | - | - | - |
| 940-941 | - | - | - | - | - | AT | - | - | AT | - | - | - | - | - | - | - | - | - | - | - | - | - | - | - | - | - | - | - | - | - | - | - | - | - | - | - | - | - | - | - | - |
| 942-945 | - | - | - | - | - | CGCC | - | - | TAAT | - | - | - | - | - | - | - | - | - | - | - | - | - | - | - | - | - | - | - | - | - | - | - | - | - | - | - | - | - | - | - | - |
| 946-947 | - | - | - | - | - | AA | - | - | AA | - | - | - | - | - | - | - | - | - | - | - | - | - | - | - | - | - | - | - | - | - | - | - | - | - | - | - | - | - | - | - | - |
| 948 | - | - | - | - | - | A | - | - | - | - | - | - | - | - | - | - | - | - | - | - | - | - | - | - | - | - | - | - | - | - | - | - | - | - | - | - | - | - | - | - | - |
| 949-960 | - | - | - | - | - | R | - | - | R | - | - | - | - | - | - | - | - | - | - | - | - | - | - | - | - | - | - | - | - | - | - | - | - | - | - | - | - | - | - | - | - |
| 987 | . | . | . | . | . | . | . | . | . | . | . | . | . | . | . | . | . | . | . | . | T | T | T | T | . | . | . | . | . | . | T | T | T | T | . | . | . | . | . | . | . |
| 993 | - | - | - | - | - | - | - | - | - | - | - | - | - | - | - | - | - | - | - | - | T | - | - | - | - | - | - | - | - | - | T | - | T | - | - | - | - | - | - | - | - |
| 999 | . | . | . | . | . | . | . | . | . | . | . | . | . | . | . | . | . | . | . | . | A | A | A | A | . | . | . | . | . | . | A | A | A | A | . | . | . | . | . | . | . |
| 1000 | . | . | . | . | . | . | . | . | . | . | . | . | . | . | . | . | . | . | . | . | . | . | . | . | . | . | . | . | . | T | . | . | . | . | . | . | . | . | . | . | . |
| 1005 | . | . | . | . | . | . | . | A | . | . | . | . | . | . | . | . | . | . | . | . | . | . | . | . | . | . | . | . | . | . | . | . | . | . | . | . | . | . | . | . | . |
| 1010 | . | . | . | . | . | . | . | . | . | . | . | . | . | . | . | . | . | . | A | A | . | . | . | . | A | A | A | A | A | A | . | . | . | . | . | . | . | A | A | A | . |
| 1042 | . | . | . | . | . | . | . | . | . | . | . | . | C | C | C | C | C | C | C | C | C | C | C | C | C | C | C | C | C | C | C | C | C | C | C | C | C | C | C | C | C |
| 1080 | . | . | . | A | . | . | . | . | . | . | . | . | . | . | . | . | . | . | . | . | . | . | . | . | . | . | . | . | . | . | . | . | . | . | . | . | . | . | . | . | . |
| 1147 | . | . | . | . | . | . | . | . | . | . | . | . | T | T | . | . | . | . | . | . | . | . | . | . | . | . | . | . | . | . | . | . | . | . | . | . | . | . | . | . | . |
| 1152 | . | . | . | . | . | . | . | . | . | . | . | . | . | . | . | . | . | . | . | . | A | A | A | A | . | . | . | . | . | . | A | A | A | A | . | . | . | . | . | . | . |
| 1230 | . | . | . | . | . | . | . | . | . | . | . | . | . | . | . | . | . | . | C | C | . | . | . | . | C | C | C | C | C | C | . | . | . | . | . | . | . | C | C | C | . |
| 1252 | . | . | . | . | . | . | . | . | . | . | . | . | . | . | . | . | . | . | . | . | T | T | T | T | . | . | . | . | . | . | T | T | T | T | . | . | . | . | . | . | . |
| 1257 | . | . | . | . | . | . | . | . | . | T | T | T | . | . | . | . | . | . | . | . | . | . | . | . | . | . | . | . | . | . | . | . | . | . | . | . | . | . | . | . | . |
| 1280 | . | . | . | . | . | . | . | . | . | . | . | . | . | . | . | . | . | . | . | C | . | . | . | . | . | . | . | . | . | . | . | . | . | . | . | . | . | . | . | . | . |
| 1309 | . | . | . | . | . | . | . | . | . | . | . | . | C | C | . | . | . | . | . | . | . | . | . | . | . | . | . | . | . | . | . | . | . | . | . | . | . | . | . | . | . |
| 1318 | . | . | . | . | . | . | . | . | . | . | . | . | . | . | . | . | . | . | . | . | C | C | C | C | . | . | . | . | . | . | C | C | C | C | . | . | . | . | . | . | . |
| 1383 | . | . | . | . | . | . | . | . | . | . | . | . | . | . | . | . | . | . | G | G | G | G | G | G | G | G | G | G | G | G | G | G | G | G | . | . | . | G | G | G | . |
| 1388 | . | . | . | . | . | . | . | . | . | . | . | . | . | . | . | . | . | . | . | . | . | . | . | . | A | . | . | . | . | . | . | . | . | . | . | . | . | . | . | . | . |
| 1403 | . | . | . | . | . | . | . | . | . | . | . | . | . | . | . | . | . | . | . | . | . | . | . | . | . | . | . | . | . | . | C | . | . | . | . | . | . | . | . | . | . |
| 1421 | . | . | . | . | . | . | . | . | . | . | . | . | . | . | . | . | . | . | . | . | . | A | A | . | . | . | . | . | . | . | . | . | . | . | . | . | . | . | . | . | . |
| 1430 | . | . | . | . | . | . | . | . | . | . | . | . | . | . | . | . | . | . | C | C | C | C | C | C | C | C | C | C | C | C | C | C | C | C | . | . | . | C | C | C | . |
| 1441 | . | . | . | . | . | . | . | . | . | . | . | . | . | . | A | A | A | A | A | A | A | A | A | A | A | A | A | A | A | A | A | A | A | A | A | A | . | A | A | A | A |
| 1444 | . | . | . | . | . | . | . | . | . | . | . | . | . | . | . | . | . | . | . | . | T | T | T | T | . | . | . | . | . | . | T | T | T | T | . | . | . | . | T | . | . |
| 1459 | . | . | . | . | . | . | . | . | . | . | . | . | T | T | T | T | T | T | T | T | T | T | T | T | T | T | T | T | T | T | T | T | T | T | T | T | T | T | T | T | T |
| 1467-1468 | - | - | CA | - | - | - | - | - | - | - | - | - | - | - | - | - | - | - | - | - | TC | TA | TC | TC | - | - | - | - | - | - | TC | TC | TC | TC | - | - | - | - | - | - | - |
| 1469-1472 | - | - | - | - | - | - | - | - | - | - | - | - | - | - | - | - | - | - | - | - | S | S | S | S | - | - | - | - | - | - | S | S | S | S | - | - | - | - | - | - | - |
| 1473-1476 | - | - | Q | - | - | - | - | - | - | - | - | - | - | - | - | - | - | - | - | - | Q | Q | Q | Q | - | - | - | - | - | - | Q | Q | Q | Q | - | - | - | - | - | - | - |
| 1478 | . | . | . | . | . | . | . | . | . | . | . | . | . | . | . | . | . | . | T | T | T | T | T | T | T | T | T | T | T | T | T | T | T | T | . | . | . | T | T | T | T |
